# Draft Genome Sequence of *Tokyovirus*, a Member of the Family *Marseilleviridae* Isolated from the Arakawa River of Tokyo, Japan

**DOI:** 10.1128/genomeA.00429-16

**Published:** 2016-06-09

**Authors:** Masaharu Takemura

**Affiliations:** Laboratory of Biology, Department of Liberal Arts, Faculty of Science, Tokyo University of Science, Shinjuku, Tokyo, Japan

## Abstract

Members of the *Marseilleviridae* family are large DNA viruses with icosahedral particles that infect *Acanthamoeba* cells. This report presents a new *Marseilleviridae* family member discovered in a water/soil sample from a river in Tokyo, named *Tokyovirus*, with genome size of 370 to 380 kb.

## GENOME ANNOUNCEMENT

The *Marseilleviridae* family is a group of giant viruses with smaller particles of 200-nm diameter and with a genome size of 300 to 400 kb (1–9). This article reports the draft genome of a new virus belonging to the family *Marseilleviridae*, isolated from the coast of the Arakawa River, located in Tokyo, Japan. It was named *Tokyovirus* according to the rules of nomenclature for *Marseilleviridae*.

Water/soil samples were collected from a bank of the Arakawa River. After mud was removed by filtration (filter paper 43; Whatman PLC), samples were further filtered (0.8-µm pore size, Millex-AA; Millipore). Filtered samples were concentrated by polyethylene glycol precipitation overnight at 4°C, followed by centrifugation at 1,500 × *g* for 30 min at 4°C (10). After the supernatant was removed, the pellet was resuspended in 4 ml of peptone yeast extract-glucose (PYG) broth and then filtered again (Millex-AA; Millipore). Then, 4 ml of fresh PYG and a 1-ml suspension of amoeba cells were added to this viral solution, which was divided and cultured on 56 wells in a 96-well culture plate at 26°C. After 10 days, I found that amoeba cells in only 1 well out of 56 wells had delayed proliferation and had been almost round. The supernatant of the culture in this single well was inoculated to fresh amoeba cells of 3 wells in a 96-well culture plate. Almost all cells were rounded. The supernatant was inoculated to fresh amoeba cells in a 25 cm^2^ culture flask. After 2 days, rounded amoeba cells were harvested. Then, the supernatant was stored at 4°C as an isolated virus solution. After virus cloning (11), genomic DNA of *Tokyovirus* (1.1 µg) was prepared from PYG culture media including viral particles using NucleoSpin Tissue (Macherey-Nagel GmbH and Co. KG) according to the manufacturer’s protocol. A DNA library for sequencing was prepared using the TruSeq Nano DNA LT library prep kit (Illumina, Inc.), and sequencing was performed on a HiSeq 2500 platform (Illumina, Inc.). Edena software was used to assemble 1,000,000 reads into 68 contigs with an average length of 5,481 nucleotides (nt) and a maximum contig length of 360,777 nt. The total length of the 68 contigs was 372,707 nt. Prediction of the coding region of the *Tokyovirus* genome was conducted using CRITICA version 1.05b and Glimmer 2 version 2.10. Prediction of tRNA was conducted using tRNAScan-SE version 1.23, according to the manufacturer’s protocols. The prediction of gene function was conducted using NCBI BLASTp in the NCBI NR and NCBI COG databases.

Genome analysis showed that *Tokyovirus* has a 370- to 380-kb genome. The total length of the 68 contigs was 372,707 nt, which is approximately equal to the genome sizes of other *Marseilleviridae* members (1, 2, 4, 7). This draft sequence is predicted to have 487 coding sequences (CDSs) and 2 tRNA genes (one of them is a pseudogene). Genome comparison revealed that *Tokyovirus* closely resembles *Marseillevirus* and *Melbournevirus*. *Tokyovirus* has 47 CDSs, which are predicted to be highly similar (>90% identical) with *Marseillevirus* homologs.

### Nucleotide sequence accession number.

The draft genomic sequence of *Tokyovirus* has been deposited in DDBJ/ENA/GenBank under the accession number AP017398.

## References

[1] Boyer M, Yutin N, Pagnier I, Barrassi L, Fournous G, Espinosa L, Robert C, Azza S, Sun S, Rossmann MG, Suzan-Monti M, La Scola B, Koonin EV, Raoult D 2009 Giant Marseillevirus highlights the role of amoebae as a melting pot in emergence of chimeric microorganisms. Proc Natl Acad Sci USA 106:21848–21853. doi:10.1073/pnas.0911354106. 20007369 PMC2799887

[2] Thomas V, Bertelli C, Collyn F, Casson N, Telenti A, Goesmann A, Croxatto A, Greub G 2011 Lausannevirus, a giant amoebal virus encoding histone doublets. Environ Microbiol 13:1454–1466. doi:10.1111/j.1462-2920.2011.02446.x. 21392201

[3] Lagier J-C, Armougom F, Million M, Hugon P, Pagnier I, Robert C, Bittar F, Fournous G, Gimenez G, Maraninchi M, Trape J-F, Koonin EV, La Scola B, Raoult D 2012 Microbial culturomics: paradigm shift in the human gut microbiome study. Clin Microbiol Infect 18:1185–1193. doi:10.1111/1469-0691.12023. 23033984

[4] Aherfi S, Pagnier I, Fournous G, Raoult D, La Scola B, Colson P 2013 Complete genome sequence of Cannes 8 virus, a new member of the proposed family “Marseilleviridae.” Virus Genes 47:550–555. doi:10.1007/s11262-013-0965-4. 23912978

[5] Boughalmi M, Pagnier I, Aherfi S, Colson P, Raoult D, La Scola B 2013 First isolation of a Marseillevirus in the *Diptera syrphidae Eristalis tenax*. Intervirology 56:386–394. doi:10.1159/000354560. 24157885

[6] Aherfi S, Boughalmi M, Pagnier I, Fournous G, La Scola B, Raoult D, Colson P 2014 Complete genome sequence of Tunisvirus, a new member of the proposed family *Marseilleviridae*. Arch Virol 159:2349–2358. doi:10.1007/s00705-014-2023-5. 24770845

[7] Doutre G, Philippe N, Abergel C, Claverie J-M 2014 Genome analysis of the first *Marseilleviridae* representative from Australia indicates that most of its genes contribute to virus fitness. J Virol 88:14340–14349. doi:10.1128/JVI.02414-14. 25275139 PMC4249118

[8] Doutre G, Arfib B, Rochette P, Claverie J-M, Bonin P, Abergel C 2015 Complete genome sequence of a new member of the *Marseilleviridae* recovered from the brackish submarine spring in the cassis Port-Miou Calanque, France. Genome Announc 3(6):e01148-15. doi:10.1128/genomeA.01148-15. 26607881 PMC4661300

[9] Dornas FP, Assis FL, Aherfi S, Arantes T, Abrahão JS, Colson P, La Scola B 2016 A Brazilian Marseillevirus is the founding member of a lineage in family *Marseilleviridae*. Viruses 8:76. doi:10.3390/v8030076. PMC4810266 26978387

[10] Philippe N, Legendre M, Doutre G, Couté Y, Poirot O, Lescot M, Arslan D, Seltzer V, Bertaux L, Bruley C, Garin J, Claverie J-M, Abergel C 2013 Pandoraviruses: amoeba viruses with genomes up to 2.5 Mb reaching that of parasitic eukaryotes. Science 341:281–286. doi:10.1126/science.1239181. 23869018

[11] Legendre M, Lartigue A, Bertaux L, Jeudy S, Bartoli J, Lescot M, Alempic J-M, Ramus C, Bruley C, Labadie K, Shmakova L, Rivkina E, Couté Y, Abergel C, Claverie J-M 2015 In-depth study of *Mollivirus sibericum*, a new 30,000-y-old giant virus infecting *Acanthamoeba*. Proc Natl Acad Sci USA 112:E5327–E5335. doi:10.1073/pnas.1510795112. 26351664 PMC4586845

